# Elevated CO_2_ decreases micronutrient Zn but not Fe in vegetables – evidence from a meta-analysis

**DOI:** 10.3389/fpls.2025.1509102

**Published:** 2025-07-04

**Authors:** Xiaolin Wang, Shengmin Zhang, Haichao Li, Gijs Du Laing, Monica Odlare, Jan Skvaril

**Affiliations:** ^1^ Future Energy Center, School of Business, Society and Engineering, Mälardalen University, Västerås, Sweden; ^2^ Swedish Species Information Centre, Swedish University of Agricultural Sciences, Uppsala, Sweden; ^3^ Department of Soil and Environment, Swedish University of Agricultural Sciences, Uppsala, Sweden; ^4^ Department of Green Chemistry and Technology, Ghent University, Ghent, Belgium

**Keywords:** elevated CO_2_, Zn, Fe, Selenium (Se), vegetable, micronutrient deficiency, food security

## Abstract

With carbon dioxide (CO_2_) levels continuing to rise in the coming decades and threatening agro-ecosystems worldwide, it is crucial to understand the impact of elevated CO_2_ on global food production and security. Elevated CO_2_ levels have been found to reduce micronutrients such as Zinc (Zn) and Iron (Fe) in staple crops, potentially exacerbating the already existing global micronutrient deficiency issue. However, as vegetables serve as another key source of micronutrients, it remains uncertain to what extent this negative effect on micronutrient levels also applies to them. To address this, we investigated the effects of elevated CO_2_ on Zn and Fe in vegetables using a meta-analysis. As expected, we found a significant increase (27%, 95% CI: 14–41%) in vegetable biomass production under elevated CO_2_ levels. Elevated CO_2_ (i) significantly reduced overall Zn concentration in vegetables by 8.9% (95% CI: 4–14%), while this effect was pronounced only in fruit vegetables (11%), but not in leafy and stem vegetables; (ii) consistently exhibited minimal effects on Fe concentration in vegetables. In the context of climate change with rising CO_2_ levels, these findings suggest that elevated CO_2_ could potentially exacerbate Zn deficiencies through vegetable consumption, albeit with enhanced vegetable yields. Furthermore, as the global population increasingly adopts vegetarian diets in the future, these results underscore the need for mitigation strategies to address potential future micronutrient deficiencies.

## 1 Introduction

Atmospheric carbon dioxide (CO_2_) is projected to increase up to 550 ppm by the middle of the 21^st^ century, nearly doubling the pre-industrial CO_2_ levels (Friedlingstein et al., 2023; Lan et al., 2024; Smith and Myers, 2018). Such increases in atmospheric CO_2_ concentrations have been reported to affect human nutrition by influencing global food production and altering nutrient concentrations in staple crops (Beach et al., 2019; Myers et al., 2017; Smith and Myers, 2019). An exemplification of this phenomenon is that several food crops under elevated CO_2_ levels have shown decreased mineral nutrient concentrations (Myers et al., 2015; Semba et al., 2022; Smith et al., 2017).

Globally, more than two billion people are deficient in micronutrients (Seal and Prudhon, 2007). Among the essential elements, micronutrients such as Zinc (Zn), Iron (Fe) and Selenium (Se) are particularly critical for humans due to their critical roles in numerous biological functions and human physical growth (Belay et al., 2021; Frise et al., 2022; Jones et al., 2017; Phiri et al., 2019). However, the reductions in Zn, Fe, and Se concentrations in plants induced by increased atmospheric CO_2_ levels may potentially accelerate micronutrient deficiencies for individuals who heavily depend on crops as their primary source of food. A meta-analysis encompassing 143 comparisons of edible portions of crops, including maize, rice, wheat, sorghum and field peas, revealed that elevated CO_2_ led to significant decreases in Fe and Zn concentrations across all crops except maize (Myers et al., 2014). It was estimated that an additional 175 million people in 2050 will face Zn deficiency and around 1.4 billion individuals are anticipated to experience a reduction of more than 4% in dietary Fe due to elevated CO_2_ levels (Smith and Myers, 2018). Similarly, Se concentrations also tended to decrease in rice and cucumber under elevated CO_2_ in research trials (Wang et al., 2023; Wei et al., 2021).

Aside from staple crop consumption, vegetables are highly recommended in daily diets due to their diverse range of beneficial compounds, such as vitamins, antioxidants, minerals, and dietary fiber (Dong et al., 2020). Globally, 1.2 billion tons of vegetables were produced in 2021 and the demand for vegetables is growing (FAO, 2022). Although numerous studies have shown changes in the essential nutrients Zn, Fe and Se in staple crops under elevated CO_2_, far less attention has been devoted to the effects of elevated CO_2_ concentration on vegetable growth and quality. Moreover, in climate-controlled vegetable cultivation, elevated CO_2_ has been widely adopted as an agricultural practice for enhancing plant growth (Dong et al., 2018a, 2020). Thus, understanding vegetable growth and nutrient status under elevated CO_2_ conditions is crucial for assessing the potential impacts of rising atmospheric CO_2_ concentrations on food security.

In general, increased CO_2_ concentrations tend to increase biomass production, but the effects of elevated CO_2_ on the nutrient status of vegetables are less well recognized due to the predominant focus on biomass enhancement. From experimental observations, the impact of impact of elevated CO_2_ on nutrients in vegetables varies: some experimental trials suggested that elevated CO_2_ levels could potentially reduce the Zn, Fe and Se in vegetables including sweet peppers, tomatoes and cucumbers (Dong et al., 2018c; Pinero et al., 2017; Wang et al., 2023), while other experiments showed different outcomes (Baslam et al., 2012; Dong et al., 2018b). This disparity is likely due to the heterogeneity among experimental setups and plant species. For instance, different CO_2_ enrichment facilities, such as free-air CO_2_ enrichment systems (FACE), open-top chambers (OTC) and controlled environmental conditions (CEC) have yielded varying results (Long et al., 2006; Taub et al., 2007; Wang et al., 2013). Besides, different plant species have exhibited varying responses to elevated CO_2_ conditions (Al-Hadeethi et al., 2019; Dong et al., 2020; Taub et al., 2007). As such, a systematic quantification of the effects of elevated CO_2_ on the Zn, Fe and Se in vegetables is needed. Previous similar research has pointed out a significant reduction in Zn and Fe, but with fewer observations (n=95 versus 51 for Zn; n=97 versus 49 for Fe) (Dong et al., 2018a). In contrast, another meta-analysis focused solely on biomass production without considering nutrient factors (Dong et al., 2020). Our objective was to systematically quantify the impacts of elevated CO_2_ concentrations on biomass and micronutrients (Fe, Zn and Se) in vegetables using a meta-analysis. We hypothesized that elevated CO_2_ concentrations would increase vegetable biomass production but decrease Zn, Fe and Se concentrations in vegetables.

## 2 Materials and methods

### 2.1 Database compilation

This meta-analysis is based on studies of the effects of elevated CO_2_ on the essential elements Zn, Fe and Se in common vegetables. An extensive keyword search was performed in the databases Web of Science, and the search engine Google Scholar. The keywords used were “carbon dioxide”, “CO_2_”, “Zn”, “Zinc”, “Fe”, “Iron”, “Se”, “Selenium”, “vegetable”, “salad” and the name of a specific vegetable was also employed as a keyword (search strings are listed in Supplementary Materials). The vegetables were classified as fruit vegetables, flowery vegetables, leafy vegetables, stem vegetables, and root vegetables. Fruit vegetables include bean, cucumber, eggplant, pea, pepper, squash and tomato. Flowery vegetables include artichoke, broccoli, cauliflower, and kale. Stem vegetables included celery and potato. Leafy vegetables include arugula, basil, cabbage, dill, endive, lettuce, onion, pakchoi, parsley, spinach and Swiss chard. Root vegetables include beet, carrot, radish, sweet potato and turnip. Pea or bean and potato were categorized as fruit vegetables and stem vegetables, respectively, as they are served as vegetables in certain countries (Gopalakrishnan, 2007; Pllana et al., 2018).

Predefined inclusion criteria were applied to determine the eligibility of studies for incorporation into the meta-analysis. First, the study must include experimental treatments (elevated CO_2_ concentrations at ≥550 and ≤ 1200 µmol mol^-1^) and controls (ambient CO_2_ concentrations at ≥200 and ≤ 450 µmol mol^-1^). When multiple elevated CO_2_ levels were investigated within the same study, only the outcomes from the elevated CO_2_ level of approximately double the ambient concentration were incorporated (Lam et al., 2012). Second, the study must present original research on the examination of vegetable biomass production, and Zn and/or Fe and/or Se concentrations in vegetables under elevated CO_2_ treatments. Third, the mean and sample size for experimental treatments and control groups must be reported.

The PRISMA flow chart is given in **Supplementary Figure S1** in **Supplementary Materials** to present the screening and paper selection process. The final dataset contains 433 observations from 27 studies, with 95 observations for Zn, 97 observations for Fe, 3 observations for Se and 238 observations for biomass production. Additionally, to identify influencing factors and assess potential variation of CO_2_ impacts on biomass and nutrient status in vegetables, we collected and compiled information on the vegetable types, plant tissues, CO_2_ enrichment facilities and plant growth substrate. Selected studies of the meta-analysis were presented in **Supplementary Materials**.

### 2.2 Meta-analysis

Each effect size statistic was calculated as the log-transformed response ratio (LnRR) (Hedges et al., 1999).


$$\text{LnRR}=\text{Ln}(\frac{{\bar{\text{x}}}_{1}}{{\bar{\text{x}}}_{2}})$$

Where ${\bar{\text{x}}}_{1}$ and ${\bar{\text{x}}}_{2}$ represents the mean values in the elevated CO_2_ treatments and control groups, respectively.

The variance (*v*) of each LnRR was calculated as:


$$v=\frac{{\text{SD}}_{1}^{2}}{{\text{n}}_{1}{\;\bar{\text{x}}}_{1}^{2}}+\frac{{\text{SD}}_{2}^{2}}{{\text{n}}_{2}{\;\bar{\text{x}}}_{2}^{2}}=\frac{{\text{CV}}_{1}^{2}}{{\text{n}}_{1}}+\frac{{\text{CV}}_{2}^{2}}{{\text{n}}_{2}}$$

Where *v* is the sampling variance, SD and n are the corresponding standard deviation and sampling size, respectively, and CV is the coefficient of variation.

If standard error (SE) instead of standard deviation (SD) were presented in studies, the transformation from SE to SD was performed utilizing the following mathematical equation:


$$\text{SD}=\text{SE }\times \;\sqrt{\text{n}}$$

Where n represents the sample size.

The weighting factor (w) was computed as:


$$\text{w}=\frac{1}{v}$$

The weighted response ratio (LnRR_+_) for all experiments was calculated as


$${\text{LnRR}}_{+}=\frac{\sum_{1}^{i}(w_{i}\times {\text{LnRR}}_{i})}{\sum_{1}^{i}w_{i}}$$

Where w_i_ and LnRR_i_ are the w and LnRR from the i^th^ study.

The 95% confidence interval (95%CI) for LnRR_+_ was computed as


$$95\text{\%CI}={\text{LnRR}}_{+}\pm 1.96\sqrt{\frac{1}{\sum_{1}^{i}w_{i}}}$$

The random effect model was employed to obtain the results described above with the “metafor” package in R v.4.3.2. Elevated CO_2_ effects were considered significant if the 95% confidence interval values did not overlap with zero. The effect sizes were transformed into percentages using the equation below to better illustrate the impacts of elevated CO_2_ addition:


$$\text{Effect size}\%=({\text{e}}^{{\text{LnRR}}_{+}}-1)\times 100\%$$

For empirical papers that did not present standard deviations or statistics that allow the calculation of SD, a method called “All cases” addressing missing standard deviations (SDs) through an improved LnRR_2_ and a weighted average CV, estimated from studies that do report SDs in the dataset, was adopted as described by Nakagawa et al. (2023). Briefly, a weighted average of CVs within studies was first calculated when multiple effect sizes were reported in one study. The pooled average of CVs between studies was then computed for variance calculations and the variance was used to substitute cases that lack SDs.


$${\text{LnRR}}_{2}=\text{Ln}\left(\frac{{\bar{\text{x}}}_{1}}{{\bar{\text{x}}}_{2}}\right)+\frac{1}{2}\left(\frac{{\text{CV}}_{1}^{2}}{{\text{n}}_{1}}-\frac{{\text{CV}}_{2}^{2}}{{\text{n}}_{2}}\right)$$

$$\begin{array}{c} v({\text{LnRR}}_{2})=\frac{{\left[\frac{\sum_{i=1}^{k}\left({\text{n}}_{1i}{\text{CV}}_{1i}\right)}{\sum_{i=1}^{k}{\text{n}}_{1i}}\right]}^{2}}{{\text{n}}_{1}}+\frac{{\left[\frac{\sum_{i=1}^{k}\left({\text{n}}_{2i}{\text{CV}}_{2i}\right)}{\sum_{i=1}^{k}{\text{n}}_{2i}}\right]}^{2}}{{\text{n}}_{2}}\\ +\frac{{\left[\frac{\sum_{i=1}^{k}\left({\text{n}}_{1i}{\text{CV}}_{1i}\right)}{\sum_{i=1}^{k}{\text{n}}_{1i}}\right]}^{4}}{2{\text{n}}_{1}^{2}}+\frac{{\left[\frac{\sum_{i=1}^{k}\left({\text{n}}_{2i}{\text{CV}}_{2i}\right)}{\sum_{i=1}^{k}{\text{n}}_{2i}}\right]}^{4}}{2{\text{n}}_{2}^{2}} \end{array}$$

The details and equations for the estimators for each effect size and variance can be found in the research of Nakagawa et al. (2023).

### 2.3 Statistical analysis

The title and abstract screening process was conducted using Covidence. Data from the selected studies were collected and extracted using WebPlotDigitizer software and directly from tables. We applied intercept-only multivariate meta-analysis models, setting ‘1|Observation’ as the random effect, to test whether the lnRRs significantly differed from 0. The data were subsequently categorized into subgroups based on vegetable type, plant tissue, CO_2_ enrichment facility, and plant growth substrate. For each subgroup, similar intercept-only multivariate meta-analysis models were applied to test whether their lnRRs significantly differed from 0. The meta-analysis and visualization of the results were conducted using the metafor package and ggplot package in R v.4.3.2.

## 3 Results

### 3.1 Overall effects of elevated CO_2_ on biomass and Zn, Fe and Se in vegetables

**Figure 1:**
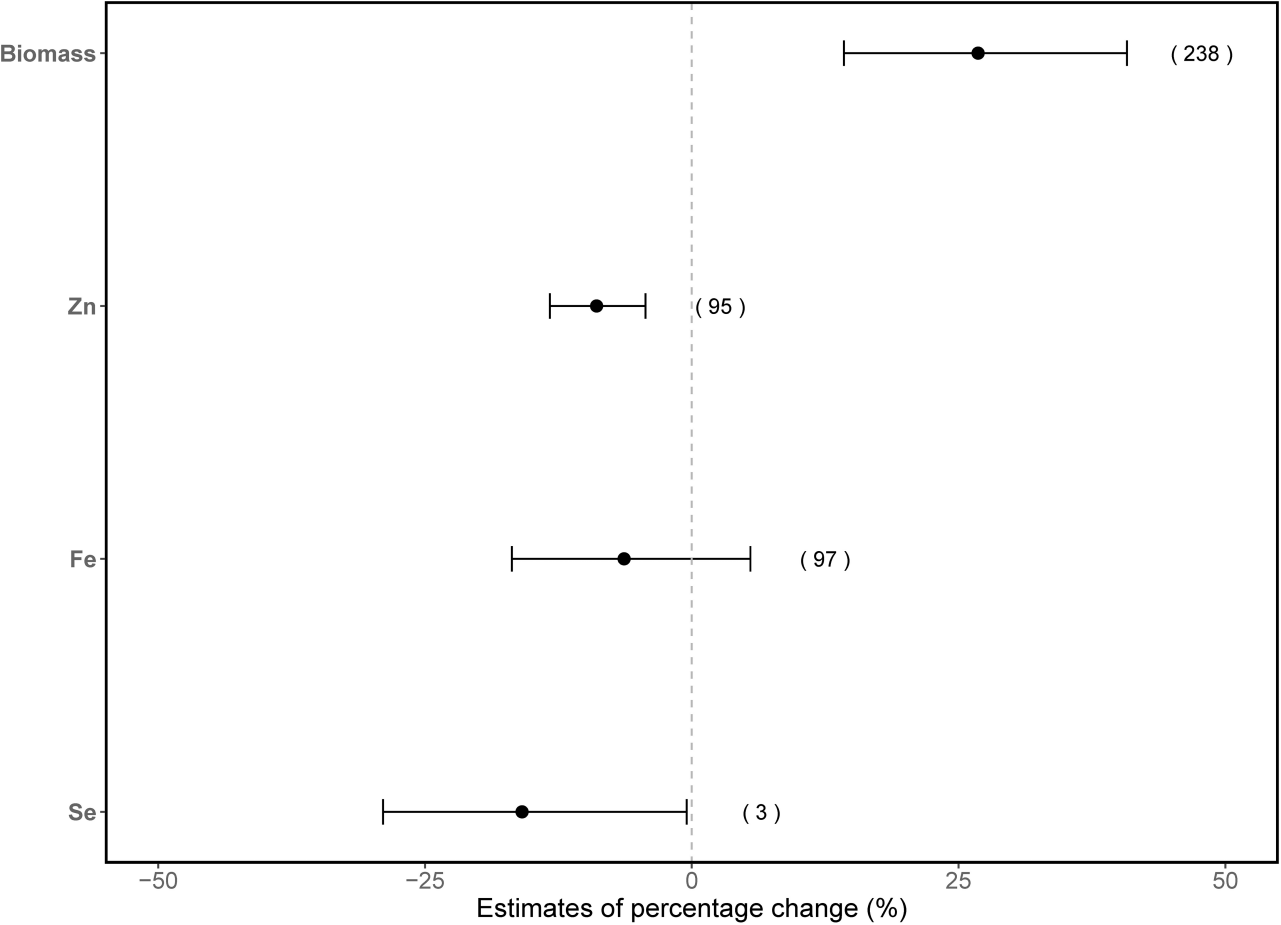
Overall effects of elevated CO_2_ on biomass production, and Zn, Fe and Se concentrations in vegetables. The x-axis values indicate estimates of percentage change with 95% confidence intervals. The numbers in parentheses represent the experimental observations of each respective indicator. Overlapping with the dashed line indicates no effect of elevated CO_2_.

Overall, elevated CO_2_ enhanced vegetable biomass production significantly by 27% (95% CI: 14–41%) (**Figure 1**). However, Zn concentration in vegetables significantly decreased by 8.9% (95% CI: 4–14%; **Figure 1**) under elevated CO_2_. A similar trend was also found for Se concentrations in vegetables, with a significant 16% (95% CI: 0.5%–29%) reduction. However, this finding for Se should be interpreted with caution due to the limited number of studies available, which may affect the robustness of the estimated effect size. In contrast, no significant effect from elevated CO_2_ on Fe concentrations was detected.

### 3.2 Variation of elevated CO_2_ effects on vegetables from different subgroups

**Figure 2:**
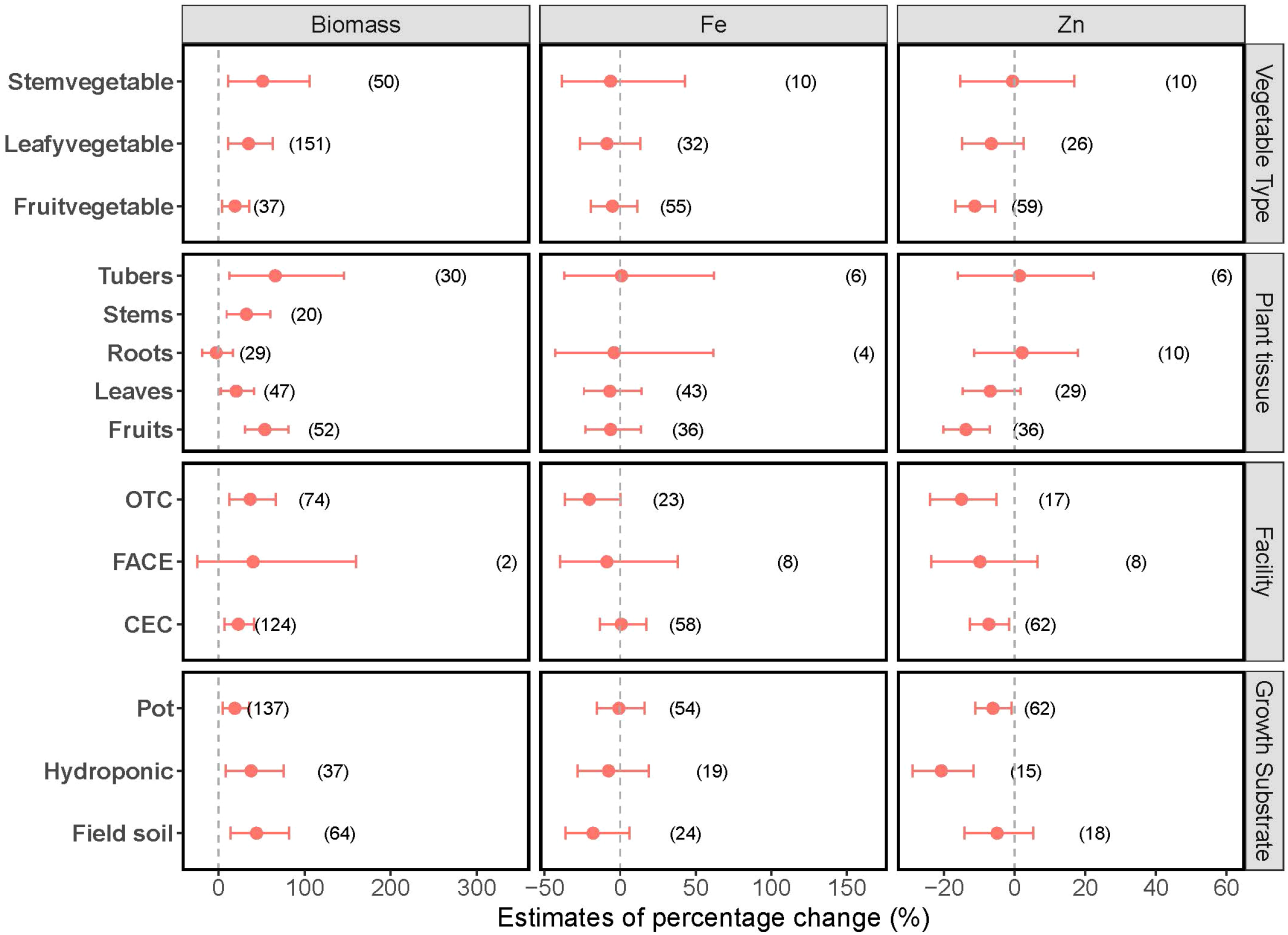
Variation in the effects of elevated CO_2_ on biomass production and Zn and Fe concentrations in vegetables across different subgroups. The x-axis values indicate estimates of percentage change with 95% confidence intervals. The numbers represent in parentheses the experimental observations of each respective indicator. Overlapping with the dashed line indicates no effect of elevated CO_2_.

A consistent positive effect of elevated CO_2_ on biomass production was observed across different vegetable groups (**Figure 2**), with significant 19%, 35% and 51% increases in biomass production for fruit vegetables, leafy vegetables, and stem vegetables, respectively. The response of biomass to elevated CO_2_ exhibited variations based on the plant tissue classification. The increase in biomass production was 54%, 20%, 32% and 66%, respectively, for fruit, leaves, stems and tubers of vegetables. In contrast, vegetable root biomass did not exhibit any changes. Moreover, the impacts of CO_2_ varied depending on CO_2_ enrichment technologies applied in agricultural practices. The biomass production of vegetables grown in controlled environmental conditions (CEC) and open-top chambers (OTC) increased by 23% and 37%, respectively, under elevated CO_2_ conditions. However, this increase was not observed with vegetables grown under free-air CO_2_ enrichment (FACE) systems, suggesting that results from controlled environments may not fully capture plant responses under field conditions. Furthermore, CO_2_ had a consistently positive effect on vegetables grown using different substrates, with 44%, 38% and 20% increases when growing in field soils, hydroponic systems and pots, respectively.

While elevated CO_2_ generally led to a decrease in Zn concentrations in vegetables, the variations manifest differently within distinct subgroups (**Figure 2**). Elevated CO_2_ resulted in a significant decrease (11%) in Zn concentration in fruit vegetables, while Zn concentrations in leafy and stem vegetables appeared unaffected. Likewise, for the fruit of vegetables, increasing CO_2_ levels led to an evident 14% reduction in Zn concentrations, while no such effect was observed for other plant parts. In studies employing CO_2_ enrichment technologies including CEC and OTC, the elevated CO_2_ induced a notable reduction of 7.3% and 15% in Zn concentrations, respectively, while FACE exhibited minimal effects. Vegetables cultivated in both hydroponic systems and pots exhibited a pronounced negative impact from elevated CO_2_ on Zn, leading to a 21% and 6.2% reduction in Zn levels, respectively. However, these effects were not observed in vegetables grown in the field at elevated CO_2_ conditions.

No significant changes in Fe concentrations were observed in vegetables under the elevated CO_2_ condition, neither overall nor within any subgroups (**Figure 2**).

## 4 Discussions

### 4.1 Effects of elevated CO_2_ on vegetable biomass

Elevated CO_2_ increases (27%) vegetable biomass significantly (**Figure 1**), which is comparable with the results from a previous meta-analysis (Dong et al., 2020) as well as the response of staple crops biomass to elevated CO_2_, with wheat increased by 23% (Ainsworth, 2008), soybean by 37% (Ainsworth et al., 2002), barley by 24% (Gardi et al., 2022) and rice by 24% (Wang et al., 2015). There are two possible explanations for the positive effects of elevated CO_2_ on plant biomass. First, elevated CO_2_ increases photosynthetic efficiency by increasing photosynthetic rate while in the meantime reducing stomatal conductance (Seneweera and Norton, 2011). A meta-analysis of 12 large-scale FACE experiments revealed that elevated CO_2_ resulted in a 31% increase in the light-saturated photosynthetic rate for 40 species (Ainsworth and Long, 2005). More specifically, elevated CO_2_ can enhance the carboxylation rate of Ribulose-1,5-bisphosphate (RuBP) carboxylase/oxygenase (RuBisCO), which is an important enzyme in plants catalyzing the initial step in the net photosynthetic CO_2_ assimilation (Spreitzer and Salvucci, 2002). Although RuBisCO exhibits a high affinity for CO_2_, it is typically not saturated at current atmospheric CO_2_ levels in C3 plants as it also binds with oxygen to catalyze the oxygenation of RuBP. However, with increased CO_2_ concentrations, the carboxylation rate of RuBisCO can be augmented, as the competitive inhibition of oxygen on RuBisCO is alleviated (Ainsworth and Long, 2005). This leads to an increase in the rate of CO_2_ fixation and the decrease in photorespiration, thereby contributing to higher photosynthetic rates and increased biomass production (Allen, 1994; Long et al., 2006, 2004). Second, biomass increase can be associated with the cultivation environment (Dong et al., 2020). Vegetables cultivated in climate-controlled environment chambers or greenhouses usually benefit from optimal growing conditions such as warmer temperature, sufficient water and nutrients. Consequently, plants exhibited greater response in terms of photosynthesis and biomass production under suitable and stable environments when elevated CO_2_ is supplied (Dong et al., 2020; Long et al., 2004). This is in agreement with our results, where a significant biomass enhancement of 23% and 37% was observed in vegetables grown in well-controlled CEC and OTC systems, respectively, while no changes were found in FACE systems.

Overall, elevated CO_2_ has a consistent positive effect on biomass production across various subgroups, with a few exceptions such as the biomass from the FACE system (**Figure 2**). This result is in line with other studies. For instance, Long et al. (2006) revealed that the enhanced yield of crops was approximately 50% less in FACE studies than in enclosure studies. One possible explanation for this lack of response in biomass in the FACE system is the co-vary factors, such as variations in temperature and precipitation fluctuations and soil heterogeneity under field conditions, influencing photosynthetic rate and biomass (Reich et al., 2014). Thus, future studies should explore the interactive effects of elevated CO_2_ with other factors on biomass production. Controlled-environment studies can provide valuable mechanistic insights, however, incorporating FACE experiments will enhance the applicability of findings to real-world scenarios.

It is important to note that while elevated CO_2_ increased biomass production for fruit, leaves, stems, and tubers of vegetables significantly, it had limited impacts on vegetable root biomass (**Figure 2**). The observed increase in tuber biomass production aligns with the findings of Miglietta et al. (2002) who reported that rising CO_2_ levels significantly enhanced tuber yield of potatoes. This difference between tubers and roots can be attributed to their distinct functions. Tubers act as storage organs where excess carbon fixed during photosynthesis is deposited as starch (Turesson, 2014). Elevated CO_2_ enhances photosynthetic activity, leading to increased carbohydrate production, which is preferentially allocated to storage tissues like tubers, thus increasing the biomass production of tubers. The unchanged root biomass aligns with the limited effect of elevated CO_2_ on root biomass previously observed in grassland ecosystems (Arnone et al., 2000) and barley (Martín-Olmedo et al., 2002). This may be attributed to the increased water use efficiency via reduced stomatal conductance under elevated CO_2_, decreasing the demand for water supply and thereby mitigating the necessity for a larger root system (Phillips et al., 2005; Reich et al., 2014). However, this result does not align with the findings that elevated CO_2_ increases root production from grassland, forest and agriculture systems (Nie et al., 2013). This discrepancy might result from the short-term exposure to rising CO_2_ levels since vegetables have shorter growth cycles compared to other staple crops and tree species. Further research is needed to verify this explanation. Nevertheless, our result suggests that the effects of elevated CO_2_ primarily manifest in non-root tissues.

### 4.2 Effects of elevated CO_2_ on Zn, Fe and Se concentration in vegetables

Aligned with our expectation, elevated CO_2_ led to an average 8.9% decrease in Zn concentrations in vegetables (**Figure 1**). Rising atmospheric CO_2_ levels have been reported to decrease mineral concentrations in staple crops. For example, a meta-analysis of the response of diverse species to elevated CO_2_ has demonstrated that Zn concentration was decreased by 9.1% in wheat, 3.4% in rice, 5.6% in soybeans and 5.2% in corn, respectively (Al-Hadeethi et al., 2019). Additionally, meta-analyses focusing on individual species further corroborated these findings, with a 3.7% decrease in Zn concentration in rice (Hu et al., 2022) and 12% in wheat (Broberg et al., 2017). Likewise, with increasing CO_2_ levels, experimental studies have observed a decreased Zn concentration in vegetables, such as tomatoes (Khan et al., 2013), potatoes (Kumari and Agrawal, 2014) and cucumbers (Dong et al., 2018b). The mechanism behind the reduction in mineral contents such as Zn associated with increasing CO_2_ levels has not been fully elucidated (Myers et al., 2014; Soares et al., 2019). “Dilution effect” has been proposed to account for this phenomenon, wherein the increased carbohydrate production leads to a decrease in mineral concentration (Jarrell and Beverly, 1981; Myers et al., 2014). This appears to explain an overall 27% increase in biomass and an 8.9% decrease in Zn concentration in our study. Besides, resulting from the reduced stomatal conductance in response to the rising CO_2_ levels, plants tend to exhibit decreased transpiration. This reduction in transpiration could result in diminished mass flow, consequently leading to reduced nutrient uptake such as the uptake of Zn (Ben Mariem et al., 2021; Li et al., 2018; McGrath and Lobell, 2013).

Across all studies in the present analysis, elevated CO_2_ decreased Zn concentration in fruit vegetables but not in leafy vegetables and stem vegetables (**Figure 2**). When it comes to different tissues of vegetables, similarly, elevated CO_2_ decreased Zn concentration in fruit tissues rather than in other parts of vegetables. This may be ascribed to the slower re-translocation of Zn within plants via phloem under elevated CO_2_, making it less easily redistributed to fruits after root uptake (Olsen and Palmgren, 2014; Page and Feller, 2015; Ujiie et al., 2019). Besides, the biomass of fruits appears to be more sensitive to elevated CO_2_ concentrations, showing the highest biomass increase (55%, **Figure 2**) compared to other plant parts, which can be partially explained by the “dilution effects” in fruits.

CO_2_ enrichment facilities also impacted the response of Zn concentration in vegetables to elevated CO_2_. Cultivating OTC and CEC systems resulted in a greater decrease in Zn compared to the FACE system. This aligns with the increased biomass production in vegetables cultivated with OTC and CEC systems under elevated CO_2_, which might be again explained as the “dilution effect”. There are also two other possible explanations. First, the variations in weather conditions such as temperature and water fluctuations in FACE system might account for limited effects of elevated CO_2_ on Zn. This can be further supported by the observed larger decrease of Zn in vegetables cultivated in pot and hydroponic systems than in field soil conditions (**Figure 2**). In pot system, the volume of soil substrate available for root exploration is more limited compared to field-grown plants. This constraint can affect plant growth and nutrient uptake, as roots in field conditions can expand freely, accessing a larger volume of nutrients. Second, edge effects in OTC and CEC systems might influence the response due to the warmer conditions induced in these systems compared to the FACE system (Taub et al., 2007). Together with the greater biomass production and Zn decrease, elevated CO_2_ showed greater effects on vegetables in OTC and CEC systems compared to FACE.

Similar to Zn, Se concentration in vegetables exhibited a decreasing trend under elevated CO_2_. However, we should note that there was a limited number of comparative observations in this study and the result must be interpreted with care. Current research on the response of Se in plants to CO_2_ fertilization in different plant species is limited and inconsistent. For instance, elevated CO_2_ increased Se concentration by 30% in cucumbers when 0.5 mg Se L^-1^ was applied, while no significant changes were observed at lower Se doses (Wang et al., 2023). Similarly, in staple crops, Se concentration in rice was decreased under elevated CO_2_ (Wei et al., 2021), while other studies did not observe any changes in Se levels in wheat (Högy et al., 2013) and soybeans (Köhler et al., 2019). These discrepancies, along with the limited studies of elevated CO_2_ effects on Se in vegetables, underscore the need for further research to validate the observed trend and clarify the underlying mechanisms.

Against our expectation, there was no significant changes in Fe concentrations in vegetables under elevated CO_2_ conditions (**Figures 1**, **2**). This finding differed from the meta-analysis by Dong et al. (2018a), who found a 16% reduction in Fe in vegetables with rising CO_2_ levels. This discrepancy can be attributed to the fewer comparison observations (n=49) included in their analysis compared with our work (n=97) since fewer observations might lead to a narrower data cope. The non-significant changes of Fe in vegetables observed in the present study also contrast with the findings reported for staple crops. For instance, in a meta-analysis, elevated CO_2_ conditions decreased around 4-6% of Fe content in staple crops (Al-Hadeethi et al., 2019). This variation in the Fe response might be attributed to the differences among the various species investigated. For example, Myers et al. (2014) also revealed that elevated CO_2_ was associated with significant decreases in Fe levels in wheat (5.5%), rice (7.3%), barley (10.5%) and soybean (4.1%), but no significant changes were observed in potato and sorghum. Nevertheless, the unchanged Fe level under increasing CO_2_ levels cannot be explained by the “dilution effect” theory. One potential explanation is that the change in Fe concentration in rising CO_2_ is smaller than the change in Zn. This stems from the distinct mass flow mechanisms governing the transport of these elements in plants, influenced by their differing solubilities. Compared to other micronutrients, such as calcium (Ca) and Zn, Fe is typically present at much lower concentrations in soil solution due to its low solubility, particularly under aerobic and alkaline conditions. As a result, Fe exhibits limited mobility via mass flow and its availability to plants is often constrained (McGrath and Lobell, 2013). Therefore, it is less likely influenced by the altered mass flow induced by elevated CO_2_. In addition, Fe availability in soil is affected by a complex interplay of factors, such as pH, organic matter, microorganisms and interactions with other nutrients (Colombo et al., 2013). For instance, higher pH in soil solutions reduces Fe availability. Elevated CO_2_ can further alter soil chemistry and microbial dynamics, potentially affecting nutrient cycling and availability (Blagodatskaya et al., 2010; Terrer et al., 2021). However, many studies on plant nutrition under elevated CO_2_ have not fully accounted for these soil-mediated processes influencing Fe availability. This gap highlights the need for more comprehensive research that integrates soil chemistry and plant physiology to better understand nutrient dynamics under elevated CO_2_ conditions. However, the above explanations may not apply to the situation with soilless cultivated vegetables, such as those grown in hydroponic systems, where Fe is assumed to be soluble and available in hydroponic solutions. Another possible explanation for the unchanged Fe concentration is the well-fertilized cultivation conditions in vegetables grown in hydroponic systems, where nitrogen and other nutrients are typically well-supplied, reducing nutrient limitations that could constrain Fe acquisition. For example, Fe concentration in wheat was not altered under medium nitrogen levels, while it significantly decreased under low nitrogen levels when exposed to elevated CO_2_ conditions compared to ambient CO_2_ levels (Al-Hadeethi et al., 2019). As an enzymatic cofactor of nitrogen metabolism (such as nitrite and nitrate reductase), Fe plays an important role in nitrogen assimilation in plants (Borlotti et al., 2012; Nasar et al., 2022). Thus, the well-supplied nitrogen may enhance plant health and the physiological requirements of Fe as an enzymatic cofactor, possibly altering Fe uptake in plants (McGrath and Lobell, 2013). Moreover, unlike soil-based cultivation, where nitrogen can influence Fe availability by affecting soil pH, hydroponic systems offer a controlled environment where pH is relatively stabilized, minimizing variability in Fe availability. Furthermore, due to its essential role in plant growth, plants tightly regulate Fe homeostasis and respond to both Fe deficiency and Fe overload (Morrissey and Guerinot, 2009). Thus, there may be regulations in vegetables regarding Fe changes induced by elevated CO_2_ levels to maintain Fe homeostasis, but such speculation requires further investigation.

Although changes in Zn, Fe, and Se concentrations under elevated CO_2_ are discussed, the studies included in this analysis did not account for the supply of these nutrients. It is important to note that nutrient distribution and transport in plants can be influenced by the levels of Zn and Fe supply, which may affect the observed nutrient concentrations. For instance, under sufficient Zn supply, Zn is primarily absorbed through root uptake. However, under Zn-deficient conditions, root uptake and Zn remobilization from the roots, stems, and leaves to seeds can occur, as observed in rice (Sperotto, 2013). Similarly, the supply range of Se affects its response to elevated CO_2._ For instance, Se concentration in cucumbers increased by 30% under elevated CO_2_ when 0.5 mg Se L^-1^ was applied, but no significant changes were observed at lower Se doses (Wang et al., 2023). The mechanisms underlying the effects of nutrient supply on plant responses to elevated CO_2_, however, remain to be explored.

### 4.3 Implications for nutrients deficiency and future work

Dietary deficiency of Zn, Fe and Se poses a significant global public health challenge. By 2050, an additional 175 million people were estimated to become Zn deficient due to the reduced Zn concentrations in staple crops as CO_2_ levels reach 550 ppm (Smith and Myers, 2018). From our analysis, elevated CO_2_ levels did not have a substantial impact on Fe concentration in vegetables. This finding suggests that elevated CO_2_ levels may not further exacerbate Fe deficiency stemming from vegetable consumption. However, a significant reduction (8.9%) in Zn has been observed in vegetables under rising atmospheric CO_2_ levels. This implies that rising CO_2_ levels have the potential to further exacerbate Zn deficiency related to vegetable consumption, particularly among the population who consume little animal flesh or animal-based products. Plant-based foods such as vegetables generally contain a lower Zn content compared to meat (Gibson, 2012) and the presence of inhibitors such as phytates will further impede Zn acquisition (Hunt, 2003). Thus, individuals who exclusively rely on plant-based food may face an increased risk of Zn deficiency under rising CO_2_ levels due to the reduced Zn concentration. For non-vegetarians, meat can be an important source of essential nutrients such as Zn, Fe and Se (Czerwonka and Tokarz, 2017; Gerber et al., 2009). For instance, animal-based food provides more than 50% of the Zn in adult diets in the United States, with beef alone contributing more than 25% of all Zn intake (Subar et al., 1998). Thus, individuals who can benefit from meat consumption may be less affected by the reduced Zn concentration in staple crops and vegetables under rising CO_2_ levels. However, meat consumption varies significantly from region to region. For instance, the highest levels for unprocessed red meat consumption range from 60 g to 91 g per day in Latin America and Europe, while the lowest levels range from 7 g to 34 g per day in Asia and Africa (Micha et al., 2015). Those with limited access to meat may still experience Zn deficiency due to reduced Zn concentrations in vegetables and staple crops under rising CO_2_ levels.

Se deficiency and its associated prevalence have been reported in many parts of the world, such as sub-Saharan Africa (Phiri et al., 2019), China (Chen, 2012) and Germany (Moghaddam et al., 2020), and advocating for improving the Se supply by dietary or supplemental measures has been suggested. Albeit limited observations, our results show elevated CO_2_ decreases Se concentrations significantly in vegetables. Therefore, under future climate change scenarios with rising CO_2_ levels, further research with a larger sample size is necessary to verify the trends observed.

Elevated CO_2_ showed greater effects on vegetables (higher biomass production and greater Zn decreases) cultivated in OTC and CEC systems compared to FACE. This disparity likely derives from the variation of other factors under field conditions. Moreover, given limited observations under field conditions, future field research that resembles realistic environmental conditions is needed. Besides, future work should also investigate long-term effects and consider other essential nutrients. Our current understanding is also hampered by the fact that the data available is that the available data is limited to C3 vegetables. Given the physiological differences between C3 and C4 plants in terms of carbon fixation pathways and response mechanisms to environmental conditions, it is essential to assess whether similar changes in nutrients observed in C3 plants also apply to C4 vegetables.

## 5 Conclusions

Our results show that elevated CO_2_ significantly enhanced biomass production in vegetables by 27%. However, it also led to an 8.9% reduction in Zn concentrations, while Fe concentrations in vegetables were not impacted. The severity of nutrient reductions in vegetables induced by elevated CO_2_ varied with vegetable types, micronutrients, and conditions of CO_2_ enrichment facilities. These findings suggest a risk of exacerbating Zn and Se deficiencies with the consumption of vegetables under elevated CO_2_ conditions, but it appears that this issue may not be exacerbated for Fe. The findings underscore the importance of considering the nutritional implications of climate change-induced alterations in vegetable composition, and the need to mitigate potential nutrient deficiencies in vegetables, thereby promoting global food security and human health.

## Data Availability

The raw data supporting the conclusions of this article will be made available by the authors, without undue reservation. Data is made available in Figshare: https://doi.org/10.6084/m9.figshare.29370743.v1.
