# Supplementary material for: Elevated CO2 decreases micronutrient Zn but not Fe in vegetables – evidence from a meta-analysis
Source: Front Plant Sci. 2025 Jul 4;16:1509102. doi: 10.3389/fpls.2025.1509102 (PMC12271221; doi:10.3389/fpls.2025.1509102)
Supplement: Supplementary file 1 [file Table1.docx]

Supplementary Material

**Elevated CO_2_ decreases micronutrient Zn but not Fe in vegetables – Evidence from a meta-analysis**

Xiaolin Wang^1^, Shengmin Zhang^2*^, Haichao Li^3^, Gijs Du Laing^4^, Monica Odlare^1^, Jan Skvaril^1^

^1^ Future Energy Center, School of Business, Society and Engineering, Mälardalen University, 722 23 Västerås, Sweden

^2^ Swedish Species Information Centre, Swedish University of Agricultural Sciences, 750 07 Uppsala, Sweden

^3^ Department of Soil and Environment, Swedish University of Agricultural Sciences, Lennart Hjelms väg 9, 750 07 Uppsala, Sweden

^4^ Department of Green Chemistry and Technology, Ghent University, 9000 Ghent, Belgium

^*^Corresponding author: shengmin.zhang@slu.se, Swedish Species Information Center, Swedish University of Agricultural Sciences, 750 07 Uppsala, Sweden. Tel:+46 793411778

**Search strings:**

Database: Web of Science

('CO2' or 'carbon dioxide') and ('crop' or 'vegetable' or 'salad' or 'lettuce' or 'tomato' or 'basil' or 'cabbage' or 'arugula' or 'parsley' or 'spinach' or 'broccoli' or 'celery' or 'kale' or 'wheat' or 'rice' or 'maize' or 'bean*' or 'soybean' or 'peanut' or 'barley' or 'oat') and ('Zn' or 'Zinc' or 'Fe' or 'Iron' or 'Se' or 'selenium’)

2219 articles exported on 3rd May 2023

Database: Google Scholar

('CO2' OR 'carbon dioxide') AND ('crop' OR 'vegetable' OR 'salad' OR 'lettuce' OR 'tomato' OR 'basil' OR 'cabbage' OR 'arugula' OR 'parsley' OR 'spinach' OR 'broccoli' OR 'celery' OR 'kale' OR 'wheat' OR 'rice' OR 'maize' OR 'bean*' OR 'soybean' OR 'peanut' OR 'barley' OR 'oat') AND ('Zn' OR 'Zinc' OR 'Fe' OR 'Iron' OR 'Se' OR 'selenium’)

1000 articles exported on 3rd May 2023

Database: Web of Science

('CO2' or 'carbon dioxide') and ('vegetable' or 'salad' or 'lettuce' or 'tomato' or 'basil' or 'cabbage' or 'arugula' or 'parsley' or 'spinach' or 'broccoli' or 'celery' or 'kale' or 'artichoke' or 'carrot' or 'cauliflower' or 'beet' or 'cucumber' or 'endive' or 'onion' or 'pea' or 'pepper' or 'radish') and ('Zn' or 'Zinc' or 'Fe' or 'Iron' or 'Se' or 'selenium’)

548 articles exported on 15th Nov 2023

Database: Web of Science

('CO2' or 'carbon dioxide') and ('vegetable' or 'salad' or ‘turnip' or ‘sweet potato’ or ‘potato’ or ‘dill' or ‘pakchoi’ or ‘swiss chard' or ‘eggplant’ or ‘squash' ) and ('Zn' or 'Zinc' or 'Fe' or 'Iron' or 'Se' or 'selenium’)

306 articles exported on 25th Jan 2024

Studies included in review **(n = 27)**

Studies excluded **(n = 3150)**

Full text assessed for eligibility **(n = 824)**

Studies screened by title and abstract **(n = 3974)**

Studies from databases **(n = 4073)**

**Identification**

Duplicates identified **(n = 99)**

Studies excluded **(n = 797)**

Not research article (n = 128)

Not plant or vegetables studies (n = 139)

No Zn, Fe and Se parameters (n = 410)

Not elevated CO_2_ conditions (n = 56)

Insufficient or invalid data (n = 22)

Elevated CO_2_ not as fertilizer (n = 42)

**Screening**

**Included**

Fig. S1. PRISMA flow diagram illustrating the procedure employed for the selection of studies for the meta-analysis

**Selected studies**

#1 Baslam, M., Garmendia, I., & Goicoechea, N. (2012). Elevated CO_2_ may impair the beneficial effect of arbuscular mycorrhizal fungi on the mineral and phytochemical quality of lettuce. Annals of Applied Biology, 161(2), 180-191.

#2 DONG, J. L., Xun, L. I., Nazim, G., & DUAN, Z. Q. (2018). Interactive effects of elevated carbon dioxide and nitrogen availability on fruit quality of cucumber (*Cucumis sativus* L.). Journal of integrative agriculture, 17(11), 2438-2446.

#3 Dong, J., Xu, Q., Gruda, N., Chu, W., Li, X., & Duan, Z. (2018). Elevated and super elevated CO_2_ differ in their interactive effects with nitrogen availability on fruit yield and quality of cucumber. Journal of the Science of Food and Agriculture, 98(12), 4509-4516.

#4 Fangmeier, A., De Temmerman, L., Black, C., Persson, K., & Vorne, V. (2002). Effects of elevated CO_2_ and/or ozone on nutrient concentrations and nutrient uptake of potatoes. European Journal of Agronomy, 17(4), 353-368.

#5 Garmendia, I., Rashidi, S., Quezada Salirrosas, M. R., & Goicoechea, N. (2022). Atmospheric CO_2_ concentration affects the life cycle, yield, and fruit quality of early-maturing edible legume cultivars. Journal of the Science of Food and Agriculture, 102(10), 3964-3971.

#6 Giri, A., Armstrong, B., & Rajashekar, C. B. (2016). Elevated carbon dioxide level suppresses nutritional quality of lettuce and spinach. American Journal of Plant Sciences, 7(01), 246.

#7 Khan, I., Azam, A., & Mahmood, A. (2013). The impact of enhanced atmospheric carbon dioxide on yield, proximate composition, elemental concentration, fatty acid and vitamin C contents of tomato (*Lycopersicon esculentum*). Environmental monitoring and assessment, 185, 205-214.

#8 Knecht, G. N., & O'Leary, J. W. (1983). The influence of carbon dioxide on the growth, pigment, protein, carbohydrate, and mineral status of lettuce. Journal of Plant Nutrition, 6(4), 301-312.

#9 Kumari, S., & Agrawal, M. (2014). Growth, yield and quality attributes of a tropical potato variety (*Solanum tuberosum* L. cv Kufri chandramukhi) under ambient and elevated carbon dioxide and ozone and their interactions. Ecotoxicology and environmental safety, 101, 146-156.

#10 Liu, J., Peng, X., Abdelhakim, L. O. A., Fang, L., Wei, Z., & Liu, F. (2021). Carbon dioxide elevation combined with sufficient irrigation and nitrogen fertilization improves fruit quality of tomato grown in glasshouse. Archives of Agronomy and Soil Science, 67(8), 1134-1149.

#11 Mjwara, J. M., Botha, C. E. J., & Radloff, S. E. (1996). Photosynthesis, growth and nutrient changes in non‐nodulated *Phaseolus vulgaris* grown under atmospheric and elevated carbon dioxide conditions. Physiologia Plantarum, 97(4), 754-763.

#12 Parvin, S., Uddin, S., Tausz-Posch, S., Armstrong, R., Fitzgerald, G., & Tausz, M. (2019). Grain mineral quality of dryland legumes as affected by elevated CO_2_ and drought: a FACE study on lentil (*Lens culinaris*) and faba bean (*Vicia faba*). Crop and pasture science, 70(3), 244-253.

#13 Pérez-López, U., Miranda-Apodaca, J., Lacuesta, M., Mena-Petite, A., & Muñoz-Rueda, A. (2015). Growth and nutritional quality improvement in two differently pigmented lettuce cultivars grown under elevated CO_2_ and/or salinity. Scientia Horticulturae, 195, 56-66.

#14 Perez-Lopez, U., Miranda-Apodaca, J., Munoz-Rueda, A., & Mena-Petite, A. (2015). Interacting effects of high light and elevated CO_2_ on the nutraceutical quality of two differently pigmented *Lactuca sativa* cultivars (Blonde of Paris Batavia and Oak Leaf). Scientia Horticulturae, 191, 38-48.

#15 Pimenta, T. M., Souza, G. A., Brito, F. A., Teixeira, L. S., Arruda, R. S., Henschel, J. M., ... & Ribeiro, D. M. (2023). The impact of elevated CO_2_ concentration on fruit size, quality, and mineral nutrient composition in tomato varies with temperature regimen during growing season. Plant Growth Regulation, 100(2), 519-530.

#16 Reich, M., Van Den Meerakker, A. N., Parmar, S., Hawkesford, M. J., & De Kok, L. J. (2016). Temperature determines size and direction of effects of elevated CO_2_ and nitrogen form on yield quantity and quality of Chinese cabbage. Plant Biology, 18, 63-75.

#17 Singh, H., Poudel, M. R., Dunn, B. L., Fontanier, C., & Kakani, G. (2020). Effect of greenhouse CO_2_ supplementation on yield and mineral element concentrations of leafy greens grown using nutrient film technique. Agronomy, 10(3), 323.

#18 Tripp, K. E., Peet, M. M., Pharr, D. M., Willits, D. H., & Nelson, P. V. (1991). CO_2_-enhanced yield and foliar deformation among tomato genotypes in elevated CO_2_ environments. Plant physiology, 96(3), 713-719.

#19 Wang, Z., Li, D., Gruda, N. S., Zhu, C., Duan, Z., & Li, X. (2023). How to Efficiently Produce the Selenium-Enriched Cucumber Fruit with High Yield and Qualities via Hydroponic Cultivation? The Balance between Selenium Supply and CO_2_ Fertilization. Agronomy, 13(3), 922.

#20 Boufeldja, L., Boudard, F., Portet, K., Guzman, C., Morel, S., Berger, N., ... & Poucheret, P. (2023). The Impact of Elevated Atmospheric Carbon Dioxide Exposure on Magic Tomatoes’ Nutritional Health Properties. International Journal of Molecular Sciences, 24(16), 12815.

#21 Cavagnaro, T. R., Sokolow, S. K., & Jackson, L. E. (2007). Mycorrhizal effects on growth and nutrition of tomato under elevated atmospheric carbon dioxide. Functional Plant Biology, 34(8), 730-736.

#22 Jain, V., Pal, M., Raj, A., & Khetarpal, S. (2007). Photosynthesis and nutrient composition of spinach and fenugreek grown under elevated carbon dioxide concentration. Biologia plantarum, 51(3), 559-562.

#23 Jin, C. W., Du, S. T., Chen, W. W., Li, G. X., Zhang, Y. S., & Zheng, S. J. (2009). Elevated carbon dioxide improves plant iron nutrition through enhancing the iron-deficiency-induced responses under iron-limited conditions in tomato. Plant Physiology, 150(1), 272-280.

#24 Jin, J., Armstrong, R., & Tang, C. (2019). Impact of elevated CO_2_ on grain nutrient concentration varies with crops and soils: A long-term FACE study. Science of the Total Environment, 651, 2641-2647.

#25 Li, D., Li, X., Dong, J., Gruda, N. S., & Duan, Z. (2023). Warm rootzone temperature ensures the mineral concentrations in cucumber plants under elevated [CO_2_] by improving the migration pathways of mineral elements from the soil to plants. Journal of Plant Nutrition and Soil Science.

#26 Pinero, M. C., Otalora, G., Porras, M. E., Sanchez-Guerrero, M. C., Lorenzo, P., Medrano, E., & Del Amor, F. M. (2017). The form in which nitrogen is supplied affects the polyamines, amino acids, and mineral composition of sweet pepper fruit under an elevated CO_2_ concentration. Journal of agricultural and food chemistry, 65(4), 711-717.

#27 Wang, H., Fan, H., Li, Y., Ge, C., & Yao, H. (2023). Elevated CO_2_ altered the nano-ZnO-induced influence on bacterial and fungal composition in tomato (*Solanum lycopersicum* L.) rhizosphere soils. Environmental Science and Pollution Research, 1-14.
